# Cancel culture can be collectively validating for groups experiencing harm

**DOI:** 10.3389/fpsyg.2023.1181872

**Published:** 2023-07-20

**Authors:** Marissa Traversa, Ying Tian, Stephen C. Wright

**Affiliations:** Intergroup Relations and Social Justice Lab, Department of Psychology, Simon Fraser University, Burnaby, BC, Canada

**Keywords:** cancel culture, collective validation, collective action, intergroup relations, sexism, racism

## Abstract

**Introduction:**

Social psychological research on collective action and intergroup harm has yet to adequately consider the potential role of cancel culture or feelings of collective validation in motivating collective action. The current research will begin to fill this gap and may broaden our understanding of the psychological mechanisms that inspire and maintain collective action in response to intergroup harm. To our knowledge, this research is the first social psychological analysis of the impact of cancel culture on collective action and as means for producing feelings of collective validation.

**Methods:**

In two experimental studies, participants read a story describing an event of discrimination against their group followed by a manipulation of the presence or absence of an episode of cancel culture. Study 1 samples woman university students (*N* = 520) and focuses on their responses to a sexist incident on campus. Study 2 (pre-registered) assesses the generality of the model in a racism context with a community sample of East Asian Canadians and Americans (*N* = 237).

**Results:**

Study 1 showed that an episode of cancel culture had an indirect positive effect on collective action intentions mediated by feelings of collective validation and collective empowerment. Study 2 showed the indirect effect of cancel culture on collective action intentions mediated by feelings of collective validation and collective anger and contempt.

**Discussion:**

The current research offers a novel theoretical and empirical introduction to the concept of collective validation and the understudied context of cancel culture to the existing social psychological research and theory on collective action. Further, cancel culture has been criticized as problematic. However, this perspective centres those in positions of power. Through this research, we hope to shift the focus onto marginalized groups’ perspectives of episodes of cancel culture. This research shows that groups who experience harm find these episodes of cancel culture validating in ways that have yet to be fully explored by intergroup relations research. Further, these findings suggest that collective validation does mediate the relationship between cancel culture and collective action; thus, cancel culture becomes an important contributor to resistance by marginalized groups through collective validation.

## Introduction

Cancel culture involves the highly visible calling for and enacting of boycotts, condemnation, and social exiling of a person or group whose harmful behaviours or attitudes have been deemed unacceptable, offensive, or inappropriate. While predominately online, the practice of cancelling and cancel culture predates the internet and has its foundations in Black liberation and protest. Anne Charity Hudley, chair of linguistics of African America for the University of California Santa Barbara, states,

*[w]hile the terminology of cancel culture may be new and most applicable to social media through Black Twitter, in particular, the concept of being canceled is not new to Black culture* *[… cancelling is]* *a survival skill as old as the Southern black use of the boycott* *(from* *Romano, 2020**)*.

She likens cancel culture to protest and boycott of people and groups, rather than businesses, and describes cancel culture as a way to empower those whose voices are marginalized; “*it’s a collective way of saying, ‘We elevated your social status, your economic prowess, [and] we are not going to pay attention to you in the way that we once did. I may have no power, but the power I have is to [ignore] you’*” (quote from Romano, 2020).

However, cancel culture has been criticized by some as more problematic than helpful (Ronson, 2016; Hagi, 2019; Romano, 2020; see also Drury, 2002 for reactionary crowds). Equity, diversity, and inclusion (EDI) advocate and social media influencer, Beecham (2021) argues that while the goal of cancel culture is to combat prejudice, in practice it often redirects prejudice to a new target through “us vs. them” thinking. Beecham’s argument implies a singular goal of cancel culture as combatting prejudice and claims that cancel culture is ineffective in achieving this goal. It seems hasty to claim that cancel culture’s only goal – or main goal – is to combat prejudice, and conclusions about the overall value of this social practice are premature without first considering a wider range of possible goals and impacts, especially the impacts on those who have been harmed and are speaking up. This is not to say that Beecham has neglected to consider the impacts of cancel culture on harmed groups, or that all her critiques of cancel culture are or erroneous – many have merit. Rather, we propose that cancel culture, whether effective at reducing prejudice or not, may reduce the impact of harm and/or elicit other productive forms of collective action. Harmed groups may benefit from the support, validation, and visibility from others who are involved in cancelling perpetrators of harm. That is, an episode of cancel culture might provide the conditions for the harmed group to experience feelings of collective validation that, in turn, could increase feelings of empowerment that inspire or maintain collective action.

To our knowledge, this research is the first social psychological analysis of the impact of cancel culture on collective action. In addition, research on collective action in response to intergroup harm has yet to adequately consider the role of feelings of collective validation as a motivator of collective action. The current research will begin to fill this gap and in so doing may broaden our understanding of the psychological mechanisms that inspire and maintain collective responses to intergroup harm.

### Feelings of collective validation

Validation is typically understood as the recognition and affirmation of a person’s experiences and an affirmation of their feelings as legitimate. In clinical trauma therapy, practitioners have successfully used validation to support patient wellbeing and have identified feelings of validation as an important and necessary component of recovery and healing following harm (e.g., Hong and Lishner, 2016; Özeke-Kocabaş and Üstündağ-Budak, 2017). However, this research focuses on the psychopathology of trauma and practitioners’ use of trauma validation to support *individual* clients in *interpersonal* contexts. To develop the concept of *intergroup* validation, it may be valuable to consider group-based harm (discrimination, harassment, oppression, etc.) as trauma (Carter, 2007). Thus, the concept of validation might also be useful when understanding and thinking about responses to intergroup harm.

Discussions and investigations of validation within social psychological literature are scant, but there are a few. Kalkhoff (2005) describes collective validation as occurring when “*bystanders copy or refrain from challenging a lower-status actor’s deference to a higher-status actor [or] validate deferential behavior collectively by pressuring a recalcitrant lower-status actor to defer to a higher-status counterpart*” (p. 59). More simply, Kalkhoff is suggesting that the behaviours of bystanders validate social norms within groups by pressuring lower-status members to conform or by refraining from challenging the submission of those with lower-status. However, this use of group validation focuses on *intra*group relations, where members of a single group validate the actions of their own group members. Contrastingly, collective validation as an *intergroup* phenomenon would involve the feeling that one’s group and its experiences have been recognized and validated by members of other groups. Additionally, Kalkhoff’s definition describes only how validation can further marginalize those of “lower-status” and fails to recognize that validation might also occur where a “lower-status” group challenges a “higher- status” group – members of other groups may validate the harmed group, recognize the illegitimacy of the harm, and even join them to challenge the actions of the perpetrator group and demand reparations. Therefore, we can extend Kalkhoff’s conceptualization of collective validation by examining group-based harm where the harmed group challenges the perpetrator’s behaviour and demands justice.

An interesting and relevant example of validation is present in Foster et al.’s (2021) research on women’s expectations of validation from others for engaging in online collective action. They found that when women expected greater validation for their social media activism, they showed more interest in future collective action. However, validation in Foster and colleagues’ work focused on experiences of personal validation (validation of me as a person – likable, friendly, etc.). In addition, this work focuses on expectations that one will be validated for future actions. Here, we hope to expand on this by focusing specifically on actual experienced feelings of collective validation (the feeling that one’s group is being validated).

Interestingly, collective validation of group-based harm is also briefly described in the literature on collective apologies (e.g., Hornsey and Wohl, 2013), where recognition of a group’s continued suffering and commitment to redress by the perpetrator group are seen as essential to an effective apology. An effective apology should offer this kind of validation. However, while a collective apology offers validation of a group’s suffering and their responses to that suffering, we propose that collective validation following harm may be sought from groups other than the perpetrators – from a wider range of agents, including ingroup members and especially members of the superordinate category (see Turner et al., 1987) – the larger, more inclusive group that includes the harmed group, the perpetrator group, and other groups. Thus, although the validation offered by perpetrators through apologies may be important, validation by third party groups may also be particularly valuable.

Harmed groups receiving support from a third party is also explored by Simon and Klandermans’ (2001) triangular model of politicized collective identity. This model proposes three steps that lead harmed group members to become politicized and act against harm. First, group members become aware of the harm and agree that it is unwanted (awareness of shared grievances). Next, they identify an adversary to whom they attribute the harm (adversarial attribution). If this adversary does not address their harmful behaviours, the harmed group seeks to connect with members of the broader society to gain support. Although Simon and Klandermans do not explicitly consider that the participation of third parties can serve as validation, they do consider other consequences for this recruitment of a third party, such as structuring of the understanding of the conflict as one including opponents and potential allies, rather than “*bipolar in-group/out-group confrontation*” (p. 328). This broadens the meaning of collective action to include both actions aimed at opponents (or perpetrators) and action aimed at recruiting third party allies. This implies that the contributions of other groups (third parties) are desirable and valuable to disadvantaged group members. We propose that one reason for this is that third parties offer not only a strategic advantage but also psychological validation of the ingroup and its struggle. In addition, Simon and Klandermans’ ideas map nicely onto the context of cancel culture, where members of other groups within the larger society join in the action and thus evidence themselves as potential allies with the harmed group.

Finally, most discussions of validation describe validation in terms of the actions of those who are providing validation. Thus, validation is conceptualised in terms of the behaviours of others that are intended to create the conditions for members of the harmed group to *feel validated*. However, these behaviours *may or may not* produce these feelings. Thus, any action by others is only validating to the degree that the harmed group experiences it as such. Therefore, we propose that collective validation is more aptly understood as the psychological experience of those who are targeted. Thus, the current research centers the psychological experience of the harmed group by describing and measuring collective validation as the feelings of those who have been harmed.

### Cancel culture and feelings of collective validation

Okimoto and Wenzel (2008) describe how intergroup transgressions can threaten the status and power equilibrium and also threaten the validity of common values that the victim group expects are shared across the superordinate category (their community or society). The kinds of transgressions that lead to episodes of cancel culture often involve acts that threaten the victim group’s status, power and autonomy/control over their reputation (e.g., a sexist comment threatens the status and diminishes the power and autonomy of all women in that context). This is especially salient in intergroup relations where the status of the offending group may come at the expense the harmed group. Simultaneously, values the victim believes are shared broadly within society are also violated, threatening the validity of these values (e.g., the victim believes that society values women and sexist comments violate, and thus call into question, the general support of that value). According to Okimoto and Wenzel (2008), a response to this injustice would need to address both concerns if justice is to be restored. We propose that both concerns may be partially addressed by an episode of cancel culture. When a perpetrator is “called out” for their offensive actions, they are given the opportunity to take responsibility, apologise, and restore justice. However, if the perpetrator refuses, the harmed group calls on the superordinate group – society – for justice. By isolating, humiliating, and diminishing the status of the offender, cancel culture involves the superordinate group communicating to the harmed group that their status, and autonomy over their reputation, and the importance of the shared values that had been violated by the offending group are indeed secure. Therefore, cancel culture offers to the harmed group the objective conditions that may lead to feelings of collective validation, through high visibility (e.g., amplifying the voices of harmed group members), public denouncing of social norm violation (i.e., “*we agree that the sexist comment violates a norm*”), punishment of the perpetrators and calls for justice (i.e., through cancelling and public shame), and explicit support for the harmed group (e.g., “*we believe, see, hear, and agree with you*”).

Thus, cancelling of the offending group may provide the conditions that lead members of the harmed group to feel validated through the participation of the superordinate group in reaffirming and recognizing the harmed group’s status and power, and restoring shared values in defense of the harmed group. In part this is done by increasing the visibility of the offense while simultaneously decreasing the visibility of the offender, and by increasing the visibility of the harmed group’s response to the offense, thus amplifying the voices of marginalised groups.

This process reflects Banet-Weiser’s (2018) claim that online societies function through an inequality of power, where power is embodied through visibility and attention and often politicized and commodified. Attention – in the form of likes, shares, and trending hashtags – becomes a means to promote specific online content following the same socioeconomic politics of power as the offline world. When actions challenge deeper systems of oppression and power, they become undesirable and are less likely to attract attention. Banet-Weiser explains that many popular and commodified online feminist movements, for example, fail to “*challenge deep structures of inequalities*” (p. 11) and this failure makes them more palatable to those in power and, consequently, leads them to be more visible. Additionally, “for some images and practices to become visible, others [those that do challenge deep structures of inequalities] must be rendered invisible” (p. 11). Cancelling a perpetrator group or its members can, at times, amplify and make more visible harmed and marginalized groups.

Thus, visibility may be key in inspiring feelings of collective validation as it offers public recognition and the possibility that others will also affirm the suffering caused by intergroup harm. Therefore, this research uses the context of cancel culture as one that could inspire feelings of collective validation after group-based harm because it includes the following:

- It is provided by members of a superordinate group
- It directly recognizes that harm was done to a particular group
- It explicitly affirms the harmed group’s emotional responses to that harm
- It supports and amplifies the harmed group in challenging the perpetrator group’s actions and in demanding justice
- It has high public visibility

Even when limited and sporadic, episodes of cancel culture may have positive influences on social change through amplifying the harmed group’s voices, by temporarily canceling powerful and harmful voices, and by raising the visibility of marginalized groups. This explicit recognition and support (i.e., validation) of a harmed group may allow them to recognize their collective power (i.e., feeling of collective empowerment) and thus motivate continued efforts for social change. Therefore, we propose that one way that cancel culture can influence subsequent actions is through the resulting experience (or subjective feeling) of collective validation and its subsequent impact on feelings of collective empowerment.

### Collective empowerment and collective action

We propose that experiencing validation following harm can play an important role in building the sense of collective empowerment that is critical for members of disadvantaged groups to initiate, join, and continue their involvement in collective action directed at social change (e.g., Drury and Reicher, 1999). Rappaport (1987) defines collective empowerment as the phenomenon or process of being able or allowed to do something because there is control or authority over that thing. Similarly, Wright (2010) conceptualises perceived collective control as comprised of two beliefs: “(*1*) *that social change is contingent upon behavior* (i.e.*, that the situation is modifiable*) *and (2) that* [*a person’s*] *group in particular can execute the behaviors necessary to produce the desired change*” (p.864; see also Zimmerman and Rappaport, 1988).

Both representations of empowerment support the contention that *collective* empowerment includes two components. One is the belief that one’s *group* has the power to influence the social environment. However, one cannot experience these feelings of efficacy or power if one first does not first perceive the social environment as malleable to influence. That is, one must first perceive “instability” (Tajfel and Turner, 1979) in the current social environment. Therefore, collective empowerment involves perceptions of instability and collective efficacy. Thus, while collective validation is the feeling that one’s group’s experiences – especially with harm – are recognised, valued, and affirmed by a superordinate group, collective empowerment is the feeling that the social environment that generated harm can be changed (instability) and that one’s group has the power and means to change it (efficacy).

Thus, if experiencing collective validation serves to heighten feelings of empowerment – then cancel culture may lead marginalized groups to engage in collective efforts to achieve social change indirectly through the psychological mechanisms of feelings of collective validation and collective empowerment.

### Collective emotions and collective action

In addition to recognizing and condemning the harm done to the group, cancel culture might also offer legitimacy to collective emotions such as anger and contempt. Thus, the feeling of validation that results from an episode of cancel culture could also include the sense that one’s strong negative emotions about perpetrators of harm are appropriate, which in turn may strengthen the expression of emotions that are well-documented to provide one the psychological foundation for collective action – outgroup-directed anger and contempt. For example, Stürmer and Simon (2009) found that anger was a direct predictor of collective action as it provided an avenue for participants to relieve aggressive tension. Similarly, van Zomeren et al. (2008a) identify group-based anger as a unique pathway to collective action (see also Tausch et al., 2011; van Zomeren et al., 2012; van Zomeren, 2013) describing how “emotional social support validates the group-based appraisal of [an] event, which also affirms emotional responses like anger” (van Zomeren et al., 2004, p. 650). However, few of these studies focus on the combination of anger and contempt.

Anger and contempt are distinct, yet highly related emotions. Mackie et al. (2000) argue that anger – which is the emotional evaluation of someone’s *actions* – is related to “action tendencies against the triggering agent” (p. 610) or attack behaviours (e.g., violence, arguing). Contempt is the emotional evaluation of someone’s *worth* and is related to exclusionary actions (e.g., avoidance or exiling behaviour). Groups that are traditionally marginalized by oppressive systems may feel contempt for perpetrator groups following incidents of harm because of the long history of attempts to address injustices with little success. Simultaneously, these harmed groups might still hope for an end to oppression – which requires cooperation from perpetrator groups – and, thus, being angry or frustrated with the perpetrator groups for their lack of cooperation in resolving conflict.

Evident in most episodes of cancel culture is the presence of both anger and contempt where participants engage in *both* attack and exclusionary actions simultaneously. The act of attacking someone’s reputation is an anger response. Social exiling of perpetrators is a contempt response to unacceptable behaviour. This mixture of anger and contempt make cancel culture an interesting example of intergroup conflict.

Further, the participation of superordinate group members in cancelling of the perpetrators may communicate to the harmed group that their emotional responses of both anger and contempt are reasonable, merited, and valid. The resulting feeling of validation of one’s anger and contempt on behalf of their group’s experience may serve to increase these emotions, which should in turn increase their likelihood of engaging in collective action. Therefore, this current research will assess whether the subjective feelings of validation that emerges as a result of an episode of cancel culture also enhance anger and contempt which should lead to greater willingness to engage in collective action.

## Theoretical model

This theorizing results in a sequential mediational model that proposes that, following collective harm, an episode of cancel culture should elicit feelings of collective validation in the harmed group. This experience of collective validation strengthens feeling of both collective empowerment and the collective emotions of anger and contempt, which in turn lead to stronger intentions to engage in collective action. Thus, it is predicted that an episode of cancel culture will have indirect positive impacts on collective anger and contempt, feelings of collective empowerment, and collective action that are mediated by feelings of collective validation.

### The current research

**Figure 1:**
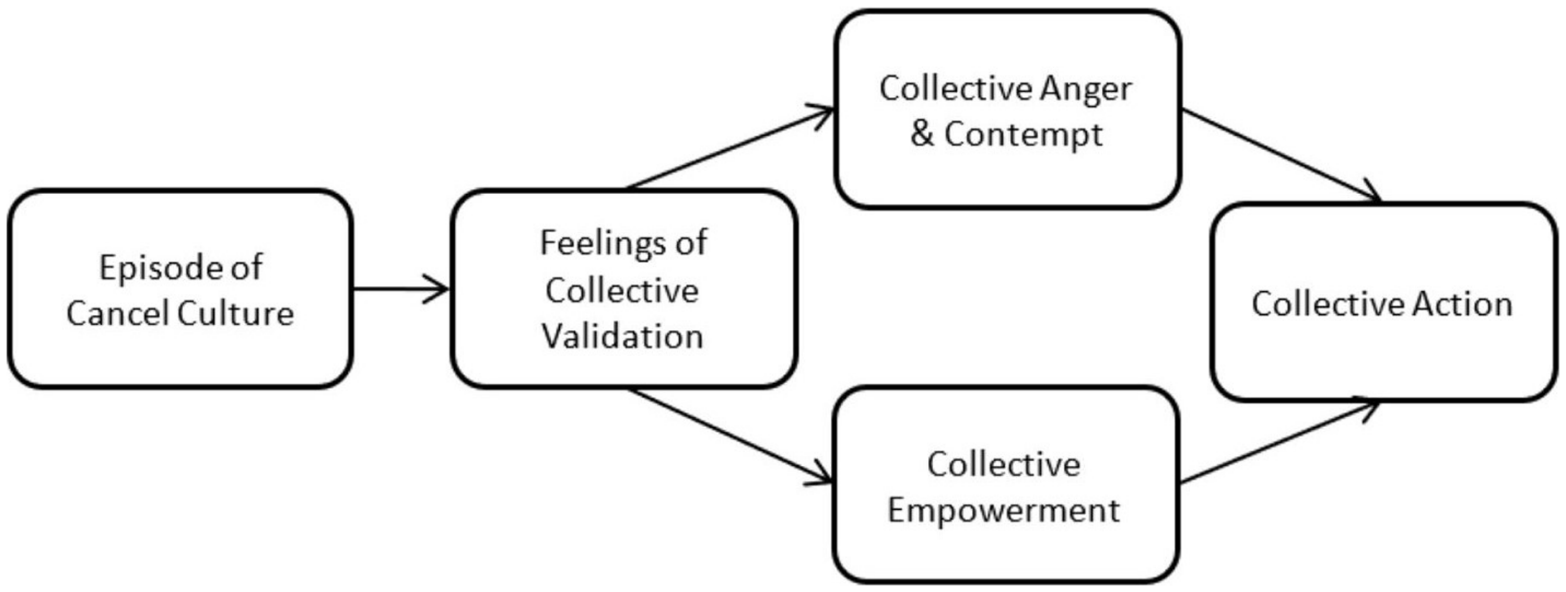
Theoretical mediation model.

Two experimental studies test the model presented in Figure 1. Study 1 uses a university student sample and focuses on the responses of women to a blatantly sexist incident on campus. Study 2 uses a community sample of East Asian Canadians and Americans and focuses on an episode of cancel culture in response to an act of anti-East Asian discrimination.

In both studies it is expected that being exposed to an episode of cancel culture, versus a control condition, will increase collective action intentions and behaviours. This effect will be sequentially mediated first by feelings of collective validation, followed by both collective anger and contempt and collective empowerment (see Figure 1). We do not have *a priori* predictions about a direct pathway between the manipulation of cancel culture and collective anger and contempt or between the manipulation of cancel culture and collective empowerment.

## Study 1: sexism and university women

### Method

#### Participants

Data were collected between October 2021 and March 2022. Cases were removed if participants did not complete the survey (133 cases), spent less than 500 s (approximately 8 min; 17 cases), or spent more than 7,200 s (120 min; 65 cases) on the survey.^1^ Finally, repeating cases were removed (20 cases). The final sample, after removing problematic cases (e.g., nonconsenting, repeated submissions, etc.), consisted of 520 university women. Demographic information is listed in Table 1.

**Table 1:** Summary of participant demographics (Study 1).

Race/ethnicity	Participants (*N* = 520)	
	*N*	%*
Black or African	9	1.7
East Asian	133	25.6
Indigenous	6	1.2
Latino/a/e or Hispanic	12	2.3
Middle Eastern/North African	34	6.5
South Asian	116	22.3
South-East Asian	54	10.4
White or European	192	36.9
Prefer to specify or provide more detail	19	3.7
Age	*N*	%
18–20	433	83.3
21–25	82	15.8
26–30	1	0.2
31–35	1	0.2
36–40	1	0.2
40+	2	0.4

* Percentage values may not equal to 100% for race/ethnicity since participants were able to select more than one category.

#### Procedure

University women were recruited online using the Psychology Department’s Research Participation System (RPS). Participants were told that the researchers were “*interested in understanding perceptions of community responses to contentious incidents and topics in campus environments*.” Those who signed up for the study were randomly assigned to one of two conditions. In both conditions, participants read a fictitious scenario of a sexist incident on campus. In the cancel culture condition this incident was followed by a description of a highly visible episode of cancel culture against the perpetrator group. In the control condition, the incident was followed by some filler/neutral information about unrelated activities on campus.

Following exposure to the given condition, participants responded to measures of feelings of collective validation, collective empowerment, anger, contempt, and collective action intentions.

##### Cancel culture condition

The cancel culture condition included a scenario describing a sexist incident on campus (modeled on a real event at Texas Tech University; see Barbato, 2014; Servantes, 2014) followed by a description of a targeted campaign by others from the community to cancel a fictitious fraternity (e.g., #EndEtaNu). The episode of cancel culture includes components naturally found in real-world cancel culture scenarios such as high visibility (“*[…] the post had 200 likes and has been shared over 30 times by the university community and beyond*”), public denouncing of norm violation (“*women on campus have every right to be pissed*”), punishment of the perpetrators (“*Two Eta Nu members […] fired from a co-op position and […] suspended from the swim team*”) and calls for justice (“*A petition […] calling for the removal of Eta Nu chapter and already has over 1,500 signatures in less than a week*”), and objective support of the harmed group (“*this is hurtful and has very real consequences for women on campus*”).

##### Control condition

The control condition included the sexist incident on campus scenario, but the description of the campaign to cancel the fraternity was replaced with filler information about other homecoming incidents unrelated to the sexist incident or to issues of gender or sexism more generally (e.g., vandalism and littering, a student getting stuck on top of a residence building).

#### Measures

##### Feelings of collective validation

This measure was constructed for this study.

Participants responded to 24 items consistent with the definition of subjective or felt collective validation previously outlined (e.g., “*Experiences of harm faced by women are recognized by the university community*”) on a 7-point scale (1 *Strongly disagree* to 7 *Strongly agree*). Higher scores indicate greater felt collective validation (*α =0*.79). See Appendix A.^2^

##### Collective anger and contempt

Participants responded to 8 items measuring anger and contempt (Mackie et al., 2000). They reported the extent to which they felt angry, displeased, irritated, furious, contemptuous, disgusted, repelled, and sick on 5-point scales (1 *Not at all* to 5 *Extremely*). Higher scores indicate greater reported feelings of anger and contempt (*α* =0.94). While Mackie et al. use two separate measures for anger and contempt, this study uses one since Mackie et al. found a high correlation between the two emotions, which was mirrored in these data (*r* = 0.86).

##### Collective empowerment

Our initial conceptualization of empowerment research included perceived instability and collective efficacy. However, our 3-item measure of perceived instability of the relationship between the perpetrator group and harmed group adapted from Mummendey et al. (1999) and Wright et al. (2020) had very low reliability (*α* = 0.56) and including it undermined the reliability of our measure of collective empowerment. Thus, these three items were not included, and the overall final measure of collective empowerment included two scales.

The second was a 5-item measure of collective efficacy adapted from Sabherwal et al. (2021). Participants rated how likely “*Women on campus, working together, can influence the following groups to do something about gender-based violence on campus*”: (a) the federal government, (b) the provincial government, (c) the local government, (d) university administrators, and (e) fraternity leaders on 7-point scales (1 *Not at all* to 7 *Extremely*). Higher scores indicate greater perceived collective efficacy (*α* = 0.85).

The third measure was a more general measure of empowerment that included 4 items measuring women’s feelings of strength, control, and power (e.g., “*I feel that women on campus are strong*”) on a 7-point agreement scale (1 *Strongly disagree* to 7 *Strongly agree*). Higher scores indicate greater feelings of empowerment (*α* = 0.81).

The collective efficacy and general empowerment measures were aggregated to provide a single collective empowerment score with higher scores indicating greater feelings of empowerment (*α* = 0.80).

##### Collective action intentions

Participants responded to 10 items measuring their willingness to participate in various forms of anti-sexism activism (e.g., “*I would participate in a rally demanding equal salaries for men and women*”; “*I would act against sexism in general*”) on 7-point scales (1 *Very unlikely* to 7 *Very likely*). This measure was adapted from Becker and Wright (2011) for use in a 2022 Canadian Context (e.g., “*I would donate for a women’s organization which lobbies for women’s rights, such as Terres des femmes*” was changed to “*I would donate to an organization that advocates for women’s rights, such as Canadian Women’s Foundation*”). Higher scores indicate stronger intentions to participate in collective action (*α* = 0.92).

## Results

### Predicted mediational analyses

The hypothesised model using the measure of Collective Action Intentions as the dependent variable was assessed using SPSS PROCESS Model 81 (Hayes, 2022).

Condition (cancel culture vs. control) had a significant direct effect on Feelings of Collective Validation, which also had a significant direct effect on Collective Empowerment. Collective Empowerment had a significant direct effect on Collective Action Intention. The direct effect of Condition on Collective Action Intentions was not significant, and the only significant indirect effect of Condition on Collective Action Intention was a sequential mediation through *both* Feelings of Collective Validation and Collective Empowerment [*β* = 0.03, 95% CI (0.01, 0.05)]. The causal steps approach to mediational analyses (see Baron and Kenny, 1986) held that a direct effect of the independent variable on the final outcome variable (or the total effect) should be a “gatekeeper” to mediational analyses. However, more recent statisticians have argued against this approach (Shrout and Bolger, 2002; Hayes, 2009) and have suggested using bootstrapping to test for indirect effects (MacKinnon et al., 2004). This bootstrapping method is the approach taken in Hayes SPSS PROCESS Macro (Hayes, 2022) that was used for these analyses. Therefore, these results suggest the need to consider other possible pathways between Condition and Collective Action Intentions that were not included in this model (Table 2).

**Table 2:** Correlation matrix for all variables.

	Mean (SD)	Condition	Collective validation	Collective emotions	Collective empowerment
Collective validation	4.3 (0.7)	0.18**			
Collective emotions	4.4 (0.7)	0.06	0.01		
Collective empowerment	4.6 (1.0)	0.09*	0.27**	−0.06	
Collective action intentions	5.6 (1.2)	−0.01	0.11*	0.26**	0.28**

* Correlation significant at the 0.05 level.

** Correlation significant at the 0.01 level.

**Figure 2:**
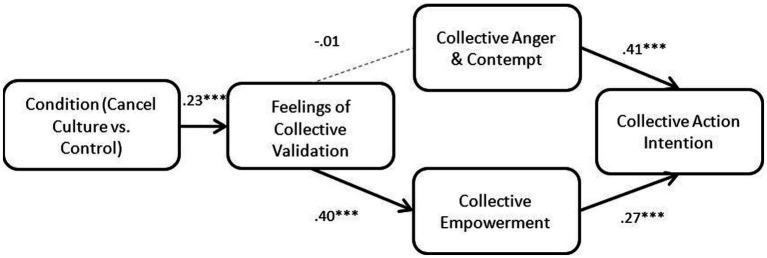
Study 1 mediation model result. Partial support for the predicted sequential indirect effects of condition on collective action intention mediated by feelings of collective validation, collective anger and contempt, collective empowerment. *p < 0.05; **p < 0.01; ***p < 0.01.

The predicted direct effect of Feelings of Collective Validation on Collective Emotions was non-significant and thus the sequential indirect effect of Condition on Collective Action Intentions mediated by Feelings of Collective Validation and Collective Emotions was non-significant (*β* = −0.00, 95% CI (−0.01, 0.01)). However, the direct effect of Collective Emotions on Collective Action Intentions was significant. See Table 3 and Figure 2 for details of these direct effects.

**Table 3:** Direct effects of predicted model 81.

	Feelings of Collective Validation				
				95% CI	
	*B*	*SE*	*p*	*LL*	*UL*
Condition	**0.23**	**0.06**	**<0.001**	**0.12**	**0.34**
Feelings of collective validation	–	–	–	–	–
Collective anger and contempt	–	–	–	–	–
Collective empowerment	–	–	–	–	–
Constant	3.92	0.09	<0.001	3.74	4.10
	*R*^2^ = 0.03 *F* (1,518) = 16.28, *p* < 0.001				
	Collective anger and contempt				
				95% CI	
	*B*	*SE*	*p*	*LL*	*UL*
Condition	0.09	0.06	0.15	−0.03	0.22
Feelings of collective validation	−0.01	0.05	0.92	−0.10	0.90
Collective anger and contempt	–	–	–	–	–
Collective empowerment	–	–	–	–	–
Constant	4.33	0.21	<0.001	3.90	4.73
	*R*^2^ = 0.00 *F* (2,517) = 1.03, *p* = 0.36				
	Collective empowerment				
				95% CI	
	*B*	*SE*	*p*	*LL*	*UL*
Condition	0.09	0.09	0.32	−0.08	0.26
Feelings of collective validation	**0.40**	**0.07**	**<0.001**	**0.27**	**0.53**
Collective anger and contempt	–	–	–	–	–
Collective empowerment	–	–	–	–	–
Constant	2.81	0.29	<0.001	2.24	3.38
	*R*^2^ = 0.07 *F* (2,517) = 20.60, *p* < 0.001				
	Collective action intent				
				95% CI	
	*B*	*SE*	*p*	*LL*	*UL*
Condition	−0.13	0.10	0.20	−0.32	0.07
Feelings of collective validation	0.10	0.08	0.32	−0.07	0.23
Collective anger and contempt	**0.41**	**0.07**	**<0.001**	**0.31**	**0.57**
Collective empowerment	**0.27**	**0.06**	**<0.001**	**0.28**	**0.54**
Constant	2.21	0.45	<0.001	1.32	3.10
	*R*^2^ = 0.14 *F* (4,515) = 21.74, *p* < 0.001				

## Discussion

This first experimental study provides initial evidence that being exposed to an episode of cancel culture does elicit feelings of collective validation and these feelings are associated with a stronger sense of collective empowerment which in turn is associated with greater collective action intention. To our knowledge, this is the first time that a positive relationship between exposure to cancel culture and intentions to engage in collective action has been demonstrated using an experimental design. However, the correlation between Condition and Collective Action Intentions is small (−0.01), implying the possible presence of other pathways in the total model. Regardless, despite legitimate critiques of cancel culture and a recognition that, like cancel culture itself, these effects may be limited in time and scope, it does appear cancel culture in response to a blatant act of sexism can have a positive impact on women’s interest in taking actions to fight gender inequality.

### Collective empowerment and feelings of collective validation

While collective – and personal – empowerment has been linked to greater collective action in previous work (Drury and Reicher, 1999; van Zomeren et al., 2008b; Wright, 2010), this research provides preliminary support for the role of collective validation as a precursor for women’s sense of collective empowerment and, thus, intentions to participate in collective action. Therefore, future research on motivating factors for collective action may benefit from the inclusion of feelings of collective validation and consider sources of this validation (e.g., cancel culture). It is also possible that instances of allyship such as cancel culture elicit collective validation of a group’s existing power through communicating collective care and, thus, group value (i.e., “*we care about your wellbeing and experiences with harm because you matter*”; e.g., Smith, 1997; Weis et al., 2006; Bradbury-Jones et al., 2011). This is not to say that cancel culture should be used to motivate groups toward collective action, rather it serves as an interesting and important example of allyship from a superordinate group that may, through collective validation (i.e., recognition, support, and increased visibility) of a harmed group, foster the collective empowerment needed to motivate groups toward collective action.

### Emotions and feelings of collective validation

Consistent with previous research (e.g., Stürmer and Simon, 2009; Tausch et al., 2011; Shepherd and Evans, 2020), stronger feelings of anger and contempt were associated with stronger collective action intentions. However, this relationship seems to be separate from cancel culture and feelings of collective validation. One may argue that the collective validation that emerges from cancel culture may alleviate feelings of anger and contempt. However, the women in the current study were substantially angry and contemptuous (*x̄* =4.43, *SD* = 0.71, on a 5-point scale). Hence, two other possible explanations for this finding are that (1) emotions constitute a pathway toward collective action that is separate from collective validation and that these emotions are more influenced by the nature of the harmful/unjust actions of the perpetrator group than by the subsequent responses to those actions and (2) there is a difference between affective social support and instrumental social support present in this research.

van Zomeren et al. (2008a) social identity model of collective action (SIMCA) proposes three distinct predictors of collective action: affective justice (emotional responses to perceived injustice – such as anger); politicized identity; and collective efficacy. Therefore, it is possible that collective anger and contempt are motivating collective action intention for reasons separate from feelings of collective validation.

In addition, according to van Zomeren et al. (2004), emotional social support (support of the harmed group’s emotions and opinions) influenced collective action through collective anger, whereas instrumental social support (direct action by a superordinate group to correct the injustice) influenced collective action through collective efficacy (see also van Zomeren et al., 2008a affective vs. nonaffective injustice). Therefore, it is also possible that the cancel culture scenario presented in the current research elicited a sense of instrumental social support more so than emotional social support resulting in the lack of a relationship between feelings of collective validation and collective anger and contempt. For example, the campus community petitioning administration to expel the perpetrators and enact consequences for the Eta Nu fraternity may have been viewed by participants as instrumental collective validation (or social support) since these examples represent public actions.

### Improvements for study 2

Study 2 will extend the current findings with three important changes from Study 1. First, we will test the generality of the findings by focusing on a different intergroup relationship. Thus, Study 2, includes a community sample of East Asian Canadians and Americans and focuses on responses to an incident of Anti-Asian discrimination. Second, in order to better consider the impact of emotions as mediators, the scenario presenting the manipulation will include more messages that reflect affective responses (van Zomeren et al., 2004). Third, to streamline the data collection, the 2-part measure of collective empowerment used in Study 1 will be replaced by a simpler, more direct measure (Moya-Garófano et al., 2021).

## Study 2: anti-East Asian racism

Incidents of violence and anti-East Asian hate crimes are on the rise following the 2020 COVID-19 outbreaks (Chen et al., 2020). In addition, the East Asian diaspora is beginning to speak openly about this xenophobia and racism. Prior to these recent events, racism toward East Asian Canadians and Americans has typically been marginalised and downplayed, at times even by East Asian communities (e.g., Tai, 2020; Shao and Lin, 2021). This may be in part due to the Model Minority Myth (MMM) that minimises and marginalizes East Asian communities and groups’ experiences with racism since they are viewed as passive, privileged, successful and even as “honorary whites” who do not complain or protest like other systemically marginalised groups (Aguirre and Lio, 2008; Yoo et al., 2010; Lee, 2022). Thus, East Asian peoples may fear that drawing attention to themselves via collective action against anti-East Asian racism might result in backlash and more racist discrimination, rather than support or solidarity, from the dominant groups that endorse the MMM. Wei et al. (2010) found that Asian American women who endorsed direct confrontations of gender discrimination reported more negative outcomes including decreased life satisfaction. While the MMM has received considerable academic and public critique, it remains a common representation of East Asian communities in both the US and Canada. The resulting lack of public attention has also led to limited attention from social psychological and anti-racism research. However, anti-Asian racism has been rampant in North America for decades, and there is growing public discourse and activism (Brockell, 2021; Government of Canada, 2021; Lee, 2022). Therefore, a focus on anti- East Asian discrimination and collective responses to it are both important and timely. Thus, we will use this context to explore the role of cancel culture, feelings of collective validation, emotions, and collective empowerment in motivating collective action.

### Focus group

Prior to beginning the study, the second author conducted a focus group with East Asian Canadian students. The rationale was to include members of East Asian communities early in the research process as we were developing the content of the manipulations and adapting the measures to this new intergroup context. It was important that the scenarios and measures be respectful of East Asian cultures, perspectives, experiences, and communities. The specific goal of the focus group was to gauge how East Asian students responded to an early draft of the scenarios used in the manipulation and to use their feedback to make alterations. In addition, it was important that the list of potential collective actions was culturally relevant (e.g., would members of East Asian communities be likely to attend a protest).

In the focus group, the students read both the control and cancel culture fictitious racism scenarios and the collective action intention measure and provided feedback on each. No changes were made to the fictitious scenarios as a result of the focus group feedback. We received positive feedback from participants about the realism, cultural sensitivity, and participant responses to both the control and cancel culture conditions. However, the collective action measure was modified to include some actions in which the group indicated members of East Asian communities would be more likely to engage (e.g., writing to government officials).

### Method

#### Participants

Data was collected via *Prolific*, an online survey research platform for social sciences. The survey was available to participants who were of East Asian descent and residing in either Canada or the United States. Participants were remunerated with $6 USD for approximately 30 min of time. Cases were removed if participants did not complete the core measures of the survey (33 cases) or spent less than 500 s (approximately 8 min; 12 cases).^3^ Despite using Prolific demographic filters, some participants did not indicate being of East Asian descent and these cases were removed (17 cases). Due to a technological error, the randomly assigned condition for two participants was not recorded by Qualtrics and these cases were removed. The total sample following removal of problematic cases consisted of 237 self-identified East Asian Canadian and American participants. Demographic information is listed in Table 4 below.

**Table 4:** Summary of participant demographics (Study 2).

Country of residence	Participants (*N* = 237)	
	*N*	%
Canada	72	30.4
The USA	165	69.6
East Asian Ethnic Group	*N*	%*
Chinese	145	61.2
Japanese	19	8
Korean	39	16.5
Mongolian	0	0
Taiwanese	16	6.8
Tibetan	1	0.4
Vietnamese	14	5.9
Prefer to specify or provide more detail	13	5.5
Parent East Asian ethnic group	*N*	%*
Chinese	148	62.4
Japanese	19	8
Korean	37	15.6
Mongolian	0	0
Taiwanese	16	6.8
Tibetan	1	0.4
Vietnamese	11	4.6
Prefer to specify or provide more detail	17	7.2
Grandparent East Asian ethnic group	*N*	%
Chinese	150	63.3
Japanese	21	8.9
Korean	38	16
Mongolian	0	0
Taiwanese	16	6.8
Tibetan	1	0.4
Vietnamese	9	3.8
Prefer to specify or provide more detail	14	5.9
Disability	*N*	%*
An autism spectrum disorder	7	3
A chronic health condition	11	4.6
A communication impairment	0	0
A developmental disability	0	0
A learning disability	4	1.7
A mental illness or health challenge	23	9.7
A mobility or orthopedic impairment	3	1.3
A sensory impairment	2	0.8
A temporary impairment due to illness or injury	3	1.3
Prefer to specify or provide more detail	1	0.4
Prefer not to answer	19	8
Not applicable	184	77.6
Gender	*N*	%
Woman (cisgender and transgender)	113	47.9
Agender	2	0.8
Non-binary/gender fluid/gender non-conforming/gender queer	2	0.8
Man (cisgender and transgender)	112	47.5
Prefer not to answer	6	2.6
Sexual orientation	*N*	%
Asexual	10	4.2
Bisexual	16	6.8
Gay	3	1.3
Lesbian	4	1.7
Pansexual	1	0.4
Heterosexual/straight	192	81
Queer	2	0.8
Prefer to self-describe	1	0.4
Prefer not to answer	8	3.4
Age (years)	*N*	%
18–24	68	28.7
25–34	88	37.1
35–44	40	16.9
45–54	24	10.1
55–64	11	4.6
65–74	1	0.4
Prefer not to answer	5	2.1
Education (highest level completed)	*N*	%
High school diploma	16	6.8
Some college	34	14.3
Undergraduate/Bachelor’s degree	139	58.6
Graduate/Master’s degree	36	15.2
Doctorate/Professional degree	9	3.8
Prefer not to answer	3	1.3
Income (yearly, pre-tax)	*N*	%
< $10,000	26	11
$10,000–$19,999	11	4.6
$20,000–$29,999	16	6.8
$30,000–$39,999	17	7.2
$40,000–$49,999	21	8.9
$50,000–$59,999	18	7.6
$60,000–$69,999	13	5.5
$70,000–$79,999	25	10.6
$80,000–$89,999	13	5.4
$90,000–$99,999	14	5.9
$100,000–$149,999	21	8.9
> $150,000	21	8.9
Prefer not to answer	20	8.5
Employment status	*N*	%
Employed full time	131	55.3
Employed part time	34	14.3
Unemployed looking for work	20	8.4
Unemployed not looking for work	1	0.4
Retired	2	0.8
Student	40	16.9
Prefer not to answer	9	3.8
Residence	*N*	%
Owned or being bought	113	47.9
Rented for money	67	28.4
Occupied without payment or money or rent	2	0.8
Student housing provided by university	4	1.7
Living with friends	2	0.8
Living with family	48	20.3

* Percentage values may not equal to 100% for race/ethnicity and disability since participants were able to select more than one category.

#### Procedure

In this pre-registered experiment,^4^ Participants were told that the researchers were interested in *“understanding responses to contentious online topics and behaviours.”* Those who signed up for the study were randomly assigned to one of two conditions. In both conditions, participants read a fictitious scenario of a racist incident in the Vancouver community. In the cancel culture condition this was followed by a description of an episode of cancel culture against the perpetrator group. In the control condition, participants read only the scenario of a racist incident without any additional information. Following exposure to the given condition, participants responded to measures of the same variables in Study 1.

**Table 5:** Correlation matrix for all variables.

	Mean (SD)	Condition	Feelings of collective validation	Collective anger and contempt	Collective empowerment
Feelings of collective validation	3.39 (0.66)	0.566**			
Collective anger and contempt	4.26 (0.82)	−0.092	0.097		
Collective empowerment	2.33 (0.91)	0.274**	0.302**	−0.382**	
Collective action intentions	3.36 (0.89)	−0.025	0.056	0.439**	−0.186**

*Correlation significant at the 0.05 level.

**Correlation significant at the 0.01 level.

**Table 6:** Direct effects of predicted model 81.

	Feelings of collective validation				
				95% CI	
	*B*	*SE*	*p*	*LL*	*UL*
Condition	**0.74**	**0.07**	**<0.001**	**0.60**	**0.88**
Feelings of collective validation	–	–	–	–	–
Collective anger and contempt	–	–	–	–	–
Collective empowerment	–	–	–	–	–
Constant	2.27	0.11	<0.001	2.04	2.49
	*R*^2^ = 0.32 *F* (1,235) = 110.76, *p* < 0.01				
	Collective anger and contempt				
				95% CI	
	*B*	*SE*	*p*	*LL*	*UL*
Condition	**−0.36**	**0.093**	**0.01**	**−0.61**	**−0.11**
Feelings of collective validation	**0.28**	**0.10**	**0.01**	**0.08**	**0.47**
Collective anger and contempt	–	–	–	–	–
Collective empowerment	–	–	–	–	–
Constant	3.86	0.28	<0.001	3.31	4.41
	*R*^2^ = 0.04 *F* (2, 234) = 5.05, *p* < 0.01				
	Collective empowerment				
				95% CI	
	*B*	*SE*	*p*	*LL*	*UL*
Condition	**0.28**	**0.14**	**0.04**	**0.01**	**0.55**
Feelings of collective validation	**0.30**	**0.10**	**<0.001**	**0.09**	**0.51**
Collective anger and contempt	–	–	–	–	–
Collective empowerment	–	–	–	–	–
Constant	0.90	0.30	<0.001	0.31	1.48
	*R*^2^ = 0.11 *F* (2, 234) = 13.96, *p* < 0.001				
	Collective action intent				
				95% CI	
	*B*	*SE*	*p*	*LL*	*UL*
Condition	0.02	0.13	0.85	−0.23	0.28
Feelings of collective validation	0.02	0.10	0.83	−0.18	0.22
Collective anger and contempt	**0.46**	**0.07**	**<0.001**	**0.32**	**0.60**
Collective empowerment	−0.03	0.07	0.65	−0.16	0.10
Constant	1.37	0.41	<0.001	0.56	2.81
	*R*^2^ = 0.19 *F* (4, 232) = 13.95, *p* < 0.001				

#### Manipulation

##### Cancel culture condition

The cancel culture condition included a scenario describing a racist incident in the community followed by a description of a targeted campaign by others from the community to cancel the perpetrator. While the racist incident described in the scenario was fictitious, it was based on real experiences of East Asian and Pacific Islander Canadians and Americans throughout the Covid-19 pandemic (e.g., Kambhampaty and Sakaguchi, 2020; Baylon and Cecco, 2021). Descriptions of these real-world experiences were reviewed prior to writing the scenario. The episode of cancel culture included components naturally found in real-world cancel culture scenarios such as high visibility (“*the post had 2,200 likes and has been shared over 300 times by the Vancouver community and beyond*”); public denouncing of norm violation (“*This is deplorable, especially during a time when we should be working together*”); punishment of the perpetrators (e.g., calls for police investigations and expulsion from programs) and calls for justice (“*It’s time for racists in this community to face the consequences of their actions*”); and expressions of support of the harmed group (“*East-Asian people are right to expect better from their neighbors*”).

##### Control scenario

The control condition included the same racist incident but did not include the description of the cancel culture episode.

### Measures

#### Feelings of collective validation

Participants responded to 24-items consistent with the definition of feelings of collective validation (e.g., “*The experiences of harm faced by East Asian people are recognized by the community*”). Responses were provided on a 5-point scale (1 *Strongly disagree* to 5 *Strongly agree*). Higher scores indicate greater felt collective validation (*α* = 0.91). See Appendix A.^5^

#### Collective anger and contempt

Participants responded to a shorter (4-item rather than 8-item) version of the anger (anger, frustration) and contempt (contemptuous and disgusted) measure adapted from Mackie et al. (2000). Responses were provided on a 5-point scale (1 *Not at all* to 5 *Extremely*). Higher scores indicate greater anger and contempt (*α* = 0.90).

#### Collective empowerment

Participants responded to a 10-item measure that asked “*As an East Asian person, if I were in the situation described in the article, I would feel*”: empowered, in control of the situation, humiliated, inferior, defenseless, full of energy, stimulated, independent, not in control of the situation, and weak (Moya-Garófano et al., 2021). Because of a technical error in the Qualtrics file, five of the 10 items (full of energy, stimulated, independent, not in control of the situation, and weak) were randomly omitted for 80 participants. Thus, a shorter 5-item version of the measure was used to maintain the entire sample. Responses were provided on a 5-point scale (1 *Not at all* to 5 *Extremely*), and the reliability for this 5-item version was good (*α* = 0.82; full 10-item measure *α* = 0.89). Scores for items indicating helplessness/disempowerment were reversed so that higher overall scores indicate greater feelings of collective empowerment.

#### Collective action intentions

Participants respond to 10-items about their willingness to participate in various forms of activism against anti-East Asian racism (e.g., “*Write a letter/email to government officials in my area regarding policies that impact East Asian peoples and cultures*”) on a 5-point scale (1 *Very unlikely* to 5 *Very likely*). This measure was adapted from Becker and Wright (2011) for use with East Asian participants but was also informed by the focus group feedback with East Asian Canadian students regarding racism in their communities and ways they would be (un)likely to respond. Higher scores indicate greater willingness/intention to participate in collective action (*α* = 0.90).

## Results

### Predicted mediational analyses

The results show a significant positive direct effect of Condition on Feelings of Collective Validation, but also a significant direct effect of Condition on Collective Empowerment that was not predicted. As predicted, there was a significant direct effect of Feelings of Collective Validation on Collective Empowerment. However, there was also a significant direct effect of Feelings of Collective Validation on Collective Anger and Contempt. In addition, there was a significant negative direct effect of Condition on Collective Anger and Contempt (not mediated by Feelings of Collective Validation) that was not predicted.

Further, Collective Anger and Contempt had a significant positive effect on Collective Action Intention, but the effect of Collective Empowerment on Collective Action Intention found in Study 1 did not emerge here.

**Figure 3:**
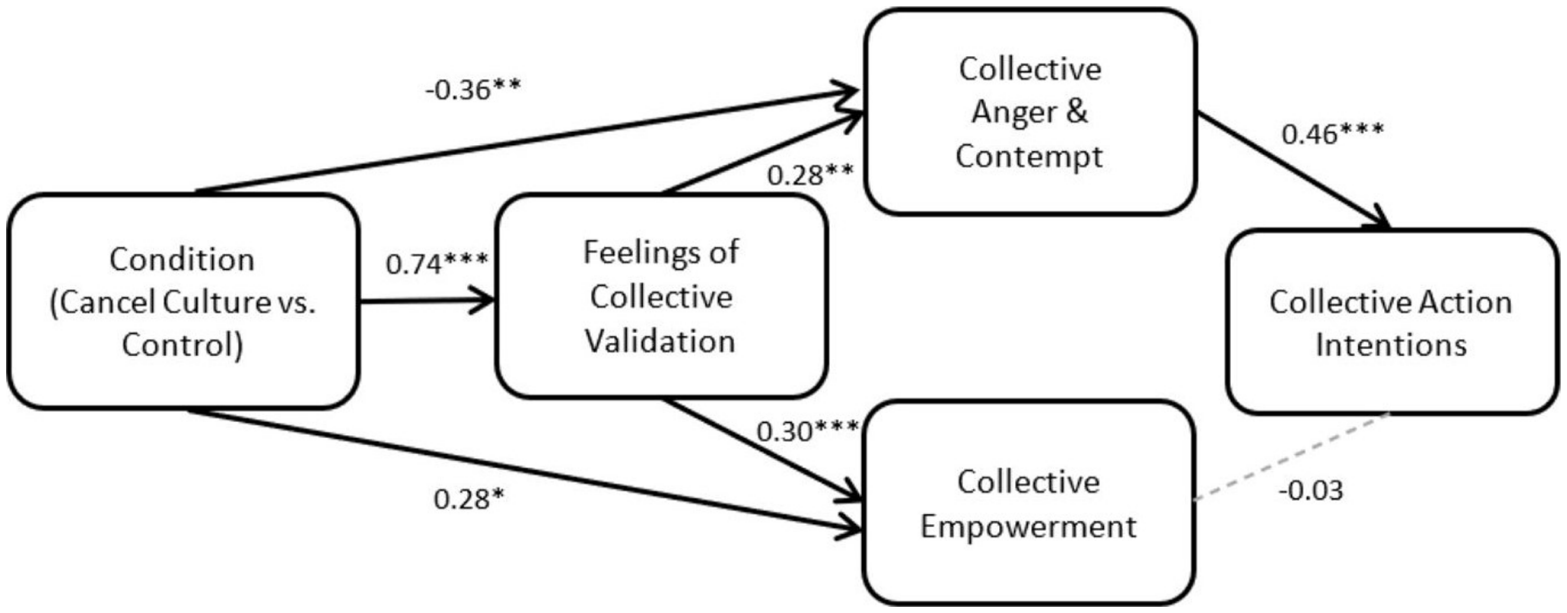
Study 2 mediation model result. Partial support for the predicted sequential indirect effects of condition on collective action intention mediated by feelings of collective validation, collective anger and contempt. The direct effects of condition on collective anger and contempt and on collective empowerment were unexpected. *p < 0.05; **p < 0.01; ***p < 0.01.

Finally, significant indirect effects were found for Condition on Collective Action Intention mediated by Collective Anger and Contempt (*β* = −0.16, 95% CI (−0.32, −0.04)), and for Condition on Collective Action Intention mediated sequentially by Collective Validation and Collective Anger and Contempt, and [*β* = 0.09, 95% CI (0.02, 0.20)]. See Tables 5, 6 and Figure 3 below for details.

## Discussion

Overall, there is support for the primary prediction that being exposed to an episode of cancel culture will elicit feelings of collective validation and that these feelings of collective validation will be positively associated with collective action intentions. However, the overall correlation between Condition and Collective Action Intentions was small, suggesting possible other variables in the total effect.

Further, it was collective validation’s positive influence on collective anger and contempt, rather than empowerment, that accounted for its positive association with collective action intentions. In fact, while exposure to an episode of cancel culture and the subsequent feelings of collective validation were associated with collective empowerment, this empowerment did not enhance collective action intentions.

### Emotions and feelings of collective validation

The significant association between Feelings of Collective Validation and Collective Anger and Contempt found in Study 2 is consistent with van Zomeren et al.’s (2004) work on affective social support versus instrumental social support. The description of the cancel culture episode in Study 2 included more evidence of shared affective experiences (e.g., it included more emotionally charged comments such as, *“If this bigot loses his business because of this, so be it*”), and this evidence of shared emotions appears to have served to validate the participants’ own feelings of collective anger and contempt. Thus, this aspect of validation may be similar to what van Zomeren et al. (2004) are describing as affective social support, which they have shown increases interest in collective action through increased anger (e.g., van Zomeren et al., 2008b). Thus, it appears that cancel culture can serve to collectively validate action-oriented negative emotions like anger and contempt if the content and tone of cancelling focuses on these shared emotions. Thus, collective validation may lead to collective action through either what van Zomeren et al. (2004) have called an emotional (usually anger; see also van Zomeren et al., 2008a) pathway or the collective empowerment pathway shown in Study 1 – or perhaps in some cases through both pathways.

Further, there was an interesting and unexpected negative direct effect of Condition on Collective Anger and Contempt and negative indirect effect of Condition on Collective Action Intentions mediated by Collective Anger and Contempt. These findings may indicate that for our East Asian participants, exposure to cancel culture in defense of their group may alleviate perpetrator-directed negative emotions such as anger and contempt, rather than increase them. Perhaps this is because, through engaging in cancel culture, the superordinate group shares the emotional burden of anger and contempt with the harmed group, especially if affective social support is present. However, these findings also emphasise the importance of the role of collective validation in the model. More research is needed to understand these effects fully.

### Collective empowerment and collective action intentions

The lack of a significant effect of empowerment on collective action intention is inconsistent with the findings of previous research and theorizing (e.g., Drury et al., 2005), as well as the surprising significant negative collective validation and empowerment pathway in the exploratory analysis. There are several possible explanations for these findings. The measure of collective empowerment only included five of the 10 items because of a random error. It is also possible that the measure did not capture the aspects of collective empowerment that drive collective action.

#### Construct validity of collective empowerment measure

The Collective Empowerment measure may be measuring something other than empowerment as it is usually defined and understood. Items on this empowerment measure such as “*humiliated*,” “*inferior*,” and “*defenseless*” may be capturing feelings of safety and group status instead of empowerment (Hartling and Luchetta, 1999; Edmondson, 2004; Farbod et al., 2017) that may lead participants to disengage in response to harmful situations to protect themselves and their communities from further harm or backlash.

To roughly assess the construct validity of the measure, Model 81 was reanalyzed using only the “*empowerment”* and “*in control of the situation”* items of the Collective Empowerment measure (α = 0.86). Both items include components of empowerment as defined by Rappaport (1987) and Wright (2010) and are consistent with the empowerment measure used in Study 1. Overall, participants reported low Collective Empowerment (*x̄* =1.95, *SD* = 0.95 on a 5-point scale). However, results show a significant, but small, *positive* direct effect of Collective Empowerment on Collective Action Intentions (*β* = 0.12, *SE* = 0.06, 95% CI (0.00, 0.25), *p* = 0.05), with small *positive* significant indirect effects of Condition on Collective Action Intentions mediated by Collective Empowerment (*β* = 0.07, 95% CI (0.00, 0.17)) and of Condition on Collective Action Intentions mediated sequentially by Feelings of Collective Validation and Collective Empowerment (*β* = 0.03, 95% CI (0.00, 0.06)). Thus, while the effects are small, there is evidence that the negative items on the Moya-Garófano et al. (2021) empowerment measure may be impacting the construct validity of the measure.

#### Feelings of collective validation and social identity

The social identity of East Asian peoples might be important for understanding the pathway from collective validation to collective action. Indeed, some prior research has identified social identity as an important and meaningful predictor of collective action. For example, van Zomeren et al.’s (2008a) SIMCA includes politicized identity as a main predictive pathway of collective action (see also Simon and Klandermans, 2001). Similarly, Foster et al. (2021) found politicized identity to be important for women engaging in online collective action. Thus, it is possible that the scenario in each study primed participants to think more about their woman (Study 1) or East Asian (Study 2) identities and this may be responsible for the differing empowerment and collective anger and contempt results.

Further, most of the literature on empowerment and collective action focuses on White Western samples and contexts (see Zimmerman, 2000; Lardier et al., 2020), which may not generalize to the East Asian Canadians/Americans in this sample. Therefore, it is possible that episodes of cancel culture that explicitly provide greater affective social support of the East Asian community (as was done in Study 2), may inspire the kind of collective validation that allows for greater expression of perpetrator-directed negative emotions. This may be, in part, because this affective social support directly challenges stereotypical expectations of East Asian people imposed by the MMM (Aguirre and Lio, 2008; Yoo et al., 2010; Lee, 2022).

Therefore, future research should consider the important role of specific social identities and the nature of the existing intergroup relations experienced by those groups. Feelings of collective validation and its impact on emotions and collective empowerment may vary depending on the specific histories, and current social realities of these different groups.

As well, it should be noted that the cancel culture scenarios in both studies were based on real-world instances and widely understood definitions of cancel culture. For example, Ng (2020) states that cancel culture is “*the withdrawal of any kind of support (viewership, social media follows, purchases of products endorsed by the person, etc.) for those who are assessed to have said or done something unacceptable or highly problematic, generally from a social justice perspective especially alert to sexism, heterosexism, homophobia, racism, bullying, and related issues*” (p. 623). The scenarios used in the current studies reflect this definition. However, it must also be recognized that no direct manipulation checks were used to assess whether participants perceived the scenarios to reflect or represent an episode of cancel culture. Thus, future studies should address this potential limitation by including manipulation checks or providing other direct evidence that participants recognized the scenarios as reflecting the core elements of cancel culture.

## General discussion

Two experimental studies examined the role of feelings of collective validation in the context of cancel culture as an important determinant of collective action through its impact on collective empowerment and collective anger and contempt. Across both studies, there is evidence that feelings of collective validation play an important mediating role in the relationship between cancel culture and collective action intentions. However, the two studies provide a somewhat less definitive story concerning the mediational processes that account for collective validation’s association with collective action. Study 1 supports only a collective empowerment pathway from collective validation to collective action intentions in a sexism context, while Study 2 supports only a collective anger and contempt pathway from collective validation to collective action intentions in an anti-East Asian racism context.

### Implications and future directions

### Cancel culture, feelings of collective validation, and collective action intentions

Overall, the present research provides novel evidence that cancel culture and feelings of collective validation should be included and examined in collective action research and theory. For example, through cancel culture, members of the superordinate group – which includes more than just members of the relevant ingroup and members of the perpetrator group – can be involved in challenging a perpetrator group’s actions and disrupting (however fleeting) the online economy of visibility and structures of inequalities. This support from other members of the superordinate group can be validating for members of the group that has been harmed and this validation can agitate them enough to challenge the individuals, groups, and systems that have perpetuated this harm.

These findings are consistent with a recent study by Foster et al. (2021, see Study 2) who found that women were motivated toward collective action when they anticipated greater personal validation from others for responding to sexist tweets. Similarly, Droogendyk et al. (2016) identify “supportive contact” as an important determinant of increased collective action by the harmed group. This concept, in which an advantaged group member explicitly expresses their opposition to inequality and supports the harmed group’s goals, coincides with the elements of cancel culture (explicitly acknowledging harm and supporting group goals). Thus, it is possible that supportive contact elicits feelings of collective validation in similar ways as cancel culture and including measures of collective validation in future work on supportive contact may offer insights into the psychological mechanisms involved in its influences on collective action.

### Cancel culture, collective validation, and Allyship

The present research also supports the inclusion of cancel culture and feelings of collective validation in research on allyship, as they appear to encompass important components of allyship. Along with supportive contact, Becker et al.’s (2022) (see also Becker and Wright, 2022) concept of “politicized contact” seems to be relevant to participation in cancel culture. In their work, Becker and colleagues show that contact that recognizes and includes discussion of group inequality is linked to greater solidarity-based allyship behaviour by advantaged group members. Thus, it seems that when members of the advantaged group, the harmed group, and even third-party groups all jointly engage, cancel culture could serve as a proxy for politicized contact and thus may increase solidarity-based collective action intentions among all three groups.

However, a critique of cancel culture is that it can backfire and alienate allies by making them afraid of being cancelled themselves for making simple mistakes. Ross (2021) claims “*[i]n our pursuit of political purity, we are alienating a lot of our allies, and we are criticizing them for not being ‘woke’ enough*.” To address this strain on the ally-ingroup relationship, Ross promotes “call in” culture where allies and group members can have open, non-judgmental conversations about harm. Ross claims that call-in culture is about “*achieving accountability with grace, love, and respect as opposed to anger, shame, and humiliation*.” This “call in” approach shares much with Becker et al.’s (2022) description of politicized contact and thus these conversations may well serve to increase allyship behaviours among the advantaged group.

However, the issue with positioning call-in culture and cancel culture against each other is that the goals and motivations of these two practices differ substantially. The goal for call-in culture, according to Ross, is to end oppression through meaningful work with allies, while one goal of cancel culture is to hold accountable powerful and perpetrator groups and people who refuse to hold themselves accountable. While calling someone in might be helpful with a willing and open ally, what happens when calling in fails because the harmful party refuses to acknowledge the harm they have caused? Who holds them responsible? How do we call in those with political and social power (e.g., celebrities, billionaires, politicians, police) who refuse to acknowledge their harmful actions? Thus, while Ross is correct in stating that the goal of the human rights movement is “*to end oppression*” and that call-in culture may be an effective method for achieving this long-term goal, it may also be true that a one-size-fits-all approach to achieving this goal is too narrow. Call-in culture may be less helpful where those who are marginalized and harmed are continuously silenced by their oppressors (e.g., as victims of sexual violence). In these situations, silencing perpetrators, prioritising support, and amplification of the harmed group seem more immediately important, especially if the harmed group deals with unique stereotypes and expectations based on their group identity (such as the MMM for East Asian communities). Therefore, cancel culture, as supported by the current research, may be effective in immediate harm-reduction for the harmed group in the form of feelings of collective validation and a subsequent stronger intention to work for change that may be another path to a long-term shift away from oppression.

## Conclusion

The current research offers a novel theoretical and empirical introduction to the concept of collective validation and the understudied context of cancel culture to the existing research and theory in the social psychological literature on collective action and related topics (e.g., allyship). We found strong support that cancel culture is collectively validating for harmed groups, and that these feelings of collective validation mediate the relationship between cancel culture and collective action intentions. Therefore, we suggest and hope that future intergroup relations research on collective action and related concepts continue to utilise collective validation and cancel culture to deepen psychological understanding of collective action motivations and various psychological outcomes for harmed groups (e.g., wellbeing and life satisfaction, collective action intention and behaviour, empowerment, group identity, etc.).

## Data availability statement

The datasets presented in this study can be found in online repositories. The names of the repository/repositories and accession number(s) can be found at: https://osf.io/qftn8/.

## Ethics statement

The studies involving human participants were reviewed and approved by Simon Fraser University Research Ethics Board. The patients/participants provided their written informed consent to participate in this study.

## Author contributions

MT: writing, editing, recruiting participants, creating materials and survey, constructing measures, main data analyses, methodology and statistical analyses plan, power analyses, and theoretical background. YT: editing, recruiting participants, creating materials, focus group, and minor data analyses. SW: editing, methodology and statistical analyses plan, creation of materials, theoretical background. All authors contributed to the article and approved the submitted version.

## Funding

This research was funded by a CGS-M SSHRC and SPSSI Clara Mayo grant awarded to the corresponding author.

## Conflict of interest

The authors declare that the research was conducted in the absence of any commercial or financial relationships that could be construed as a potential conflict of interest.

## Publisher’s note

All claims expressed in this article are solely those of the authors and do not necessarily represent those of their affiliated organizations, or those of the publisher, the editors and the reviewers. Any product that may be evaluated in this article, or claim that may be made by its manufacturer, is not guaranteed or endorsed by the publisher.

## Footnotes

^1^Participants who spent less than 500 s on the survey had more incomplete data and tended to select the same scale values throughout (e.g., selecting five for every item). Participants who spent longer than 7,200 s on the survey were more likely to spend long periods of time on one page with questions that should only have taken minutes to complete, indicating that they may have left the survey open to complete other tasks and come back to it later.

^2^See Supplementary materials for EFA.

^3^No participant spent more than 7,200 s.

^4^ osf.io/qftn8

^5^See Supplementary materials for EFA.
