# Supplementary material for: Cancel culture can be collectively validating for groups experiencing harm
Source: Front Psychol. 2023 Jul 20;14:1181872. doi: 10.3389/fpsyg.2023.1181872 (PMC10399695; doi:10.3389/fpsyg.2023.1181872)
Supplement: Supplementary file 1 [file Data_Sheet_1.docx]

Supplement A. Study 1 Materials

Manipulation

Cancel Culture Condition

**The scenario in this article is true and occurred at a Canadian University. While you read this article, imagine that this scenario occurred at Simon Fraser University.**

Fraternity House Called Out as Sexist by University Community

Members of the Eta Nu fraternity are being criticized by their university community after posting a hand-written banner reading “No means yes, yes means anal” at a homecoming party this past weekend.

The frat claims that this banner was meant to be a joke, but members of the campus community don’t find it funny. Clara Hewes, a fourth-year psychology major, states “I’ve been here for 5 years. This isn’t the first banner”. Hewes is alluding to other banners posted by the fraternity over the past decade stating similar sexually problematic messages such as “Daughter Drop off” and “Eta Nu Majors in Minors”.

Hewes received the photo of the banner from a from a friend attending the party and posted it to Twitter on Sunday in hopes of gaining support from the university community to put a stop to this behaviour. Within three days, the post had 200 likes and has been shared over 30 times by the university community and beyond. Twitter users associated with the university have responded with personal information and photos of members of the Eta Nu chapter, accompanied by calls for expulsion, suspension of the chapter, and #EndEtaNu.

“We know who you are, and you won’t get away with it” says Anthony Greyson, who is a member of the school’s lacrosse team, “Eta Nu has been doing this to students for too long. Women on campus have every right to be pissed – they have every right to demand better from this campus.”

“They hide behind the term ‘joke,’ but they don’t realize that this is hurtful and has very real consequences for women on campus” says Chhaya Banerjee, leader of the Queer Student Society on campus. Banerjee is referring to a 2012 incident in which a 20-year-old Finance and Accounting student, who wishes to remain anonymous, was sexually assaulted at a party by 24-year-old Engineering major, Andrew Cormack. “It’s time to remove Eta Nu from our campus.”

Banerjee isn’t the only student to think so. A petition has been created calling for the removal of Eta Nu chapter and already has over 1500 signatures in less than a week.

While university administration has yet to comment on their plans for the future of Eta Nu, they have been in touch with the household. Two Eta Nu members, Leo Madeira and Aren Hagopian, who are allegedly responsible for the banner, have already faced consequences – Madeira was fired from a co-op position and Hagopian was suspended from the swim team.

Control Condition

**The scenarios in this article are true and occurred at a Canadian University. While you read this article, imagine that these scenarios occurred at Simon Fraser University.**

Is Homecoming Getting Too Rowdy?

Members of the Eta Nu fraternity, a rescue of drunk accounting students, and an egregious defacing of property are on everyone’s mind after homecoming celebrations got a little too boisterous this past weekend. Most notable was the Eta Nu fraternity’s hand-written banner reading “No means yes, yes means anal” posted at one of their parties.

The frat claims that this banner was meant to be a joke – part of a long-term tradition. Clara Hewes, a fourth-year psychology major, states “I’ve been here for 5 years. This isn’t the first banner”. Hewes is alluding to other banners posted by the fraternity over the past decade stating similar sexually problematic messages such as “Daughter Drop off” and “Eta Nu Majors in Minors”. Hewes received the photo of the banner from a from a friend attending the party and posted it to Twitter on Sunday. University administration has yet to comment on their response to Eta Nu’s behaviour despite some minor backlash.

On top of that, fire rescue was called in to remove three male students who were stuck on the rooftop of their on-campus residence after a night of heavy drinking. The finance and accounting students allegedly climbed up vines and netting on the side of the three-story building to the rooftop, where they proceeded to entertain the growing crowd below by stripping down to their boxers, chanting, and trying to urinate on those passing by. Police have confirmed that the students are well aside from a pending investigation and several charges for public intoxication, defacing property, and disorderly conduct.

However, police are also hard at work investigating a public defacement of property after a white Mazda that was parked at the side of the road was flipped over and damaged by a crowd of university students attending one of these homecoming street parties. Police say that, aside from the massive amounts of litter left by the students, glass bottles and other objects were also thrown at residential houses and at police. Police are asking anyone with any information to come forward. Seven arrests have been made so far and more are expected. Police say they also responded to several noise complaints for large, out-of-control street parties with thousands of people. Twenty-six people were arrested so far for public intoxication and many more were ticketed for illegal possession of open liquor.

Supplement B.
Study 1 Feelings of Collective Validation Measure

An initial exploratory factor analysis (EFA) revealed six factors with Eigenvalues greater than one. However, after assessing the scree plot and percent of variance for each factor, only two factors were deemed relevant. Thus, a second EFA forced a 2-factor model shown below.

Table B.1. EFA for Collective Validation – Study 1.

| Items | Factor | | Possible Dimension |
| --- | --- | --- | --- |
|  | 1 | 2 | Community Support |
| I feel that the university community ignores women. (R) | **.646** | -.053 |  |
| I feel seen by the university community. | **.586** | .130 |  |
| I feel that women are being sufficiently heard by the university community. | **.752** | .00 |  |
| I feel silenced by the university community. (R) | **.519** | .199 |  |
| The experiences of harm faced by women are recognized by the university community. | **.631** | .057 |  |
| I feel that the experiences of women are not acknowledged by the university community. (R) | **.530** | .006 |  |
| The university community understands that misogyny is harmful to women. | **.667** | .060 |  |
| I feel unsupported by the university community. (R) | **.668** | .061 |  |
| I feel supported by the university community in demanding reparations. | **.659** | .169 |  |
| I feel that the outcome of the scenario was just. | **.562** | -.014 |  |
| The experiences of women are validated by the university community. | **.727** | .162 |  |
| As a woman, I feel validated by the university community. | **.748** | .126 |  |
| I feel that my emotional response to sexism and misogyny is shared by others in the university community. | .001 | **.417** | Validation of Perceptions/Stance |
| I would feel that my perception of experiences with sexism are exaggerated. (R) | -.278 | **.434** |  |
| Women are right to be angry at misogynistic men. | -.143 | **.592** |  |
| It is right to feel contempt for misogynistic men. | -.089 | **.488** |  |
| My contempt for the men in this scenario is not justified. (R) | -.162 | **.495** |  |
| My emotional response to sexism and misogyny is justified. | -.245 | .**677** |  |
| My emotional responses to sexism and misogyny are valid. | -.217 | .**559** |  |
| The experiences of harm faced by women are affirmed in the university community. | .375 | .153 | Neither factor |
| The university community response makes me feel justified in demanding reparations for harm done to women. | .118 | .256 |  |
| I feel uncomfortable demanding justice. (R) | .124 | .177 |  |
| Misogynistic men deserve to be expelled from the university community. | -.203 | .280 |  |
| I feel that my emotions about the situation were invalid. (R) | -.053 | .328 |  |

*Notes.* Principal Axis Factoring: 2 factors extracted; Varimax with Kaiser Normalization; Loadings > 0.4 are bolded.

An EFA was also done for feelings of collective validation in Study 2 and indicated a similar 2-factor model shown below.

Table B.2. EFA for Feelings of Collective validation – Study 2.

| Items | Factor | | Possible Dimension |
| --- | --- | --- | --- |
|  | 1 | 2 |  |
| I feel that this community ignores East Asian people. | **.722** | -.066 | Community Support |
| If I were in this scenario, I would feel seen by the community. | **.831** | -.005 |  |
| I feel that East Asian people are being sufficiently heard by this community. | **.778** | -.060 |  |
| If I were in this scenario, I would feel silenced by the community. | **.764** | -.088 |  |
| The experiences of harm faced by East Asian people are recognized by the community. | **.858** | -.050 |  |
| The experiences of harm faced by East Asian people are affirmed in the community. | **.719** | -.078 |  |
| I feel that the experiences of East Asian people are not acknowledged by the community. | **.759** | -.075 |  |
| This community understands that racism is harmful to East Asian people. | **.800** | -.025 |  |
| I feel that my emotional response to racism and xenophobia is shared by others in the community | **.763** | .089 |  |
| If I were in this scenario, I would feel unsupported by the community. | **.829** | -.135 |  |
| If I were in this scenario, I would feel supported by the community in demanding reparations. | **.778** | .075 |  |
| The experiences of East Asian people are validated by the community. | **.833** | -.007 |  |
| As an East Asian person, I feel validated by the community. | **.890** | .019 |  |
| If I were in this scenario, I would feel that my perception of experiences with racism are exaggerated. | .066 | **.427** | Validation of Perceptions/Stance |
| I feel that the outcome of the scenario was just. | .651 | **-.207** |  |
| East Asian people are right in their anger toward racist people. | .055 | **.744** |  |
| It is right to feel contempt for racist people. | .035 | **.657** |  |
| My contempt for the racist people in this scenario is not justified. | .050 | **.541** |  |
| My emotional response to racism and xenophobia is justified. | .111 | **.788** |  |
| My emotional response to racism and xenophobia is valid. | .024 | **.729** |  |
| The community response makes me feel justified in demanding reparations for harm done to East Asian people. | .239 | .357 | Neither factor |
| If I were in this scenario, I would feel uncomfortable demanding justice. | .196 | .254 |  |
| Racist people deserve to be expelled from the community. | .057 | .505 |  |
| If I were in this scenario, I would feel that my emotions about the situation were invalid. | .231 | .265 |  |

Notes. Principal Axis Factoring: 2 factors extracted; Varimax with Kaiser Normalization; Loadings > 0.4 are bolded.

Supplement C. Study 1 Empowerment Measure EFA

An EFA was completed for the overall empowerment measure used in Study 1 to assess the theoretical appropriateness of combining two measures into one. Results show that each measure – collective efficacy and general empowerment – all predominately loaded on separate factors and that collective efficacy accounted for most of the variance (39.22%)

Table C.1. EFA for Empowerment– Study 1.

| Item | Factor | | Measure |
| --- | --- | --- | --- |
|  | 1 | 2 |  |
| Fraternity Leaders | .337 | .149 | Collective Efficacy |
| University Administrators | **.586** | .193 |  |
| Local Government | **.906** | .033 |  |
| The Provincial Government | **.971** | -.046 |  |
| The Federal Government | **.886** | -.087 |  |
| I feel that women on campus are strong. | .000 | **.798** | General Empowerment |
| I feel that women on campus are powerful. | .000 | **.851** |  |
| As a woman on campus, I feel in control. | .169 | **.589** |  |
| As a woman on campus, I feel empowered. | .165 | **.636** |  |

*Notes:* Principal Axis Factoring; Varimax with Kaiser Normalization; Loadings > 0.4 are bolded.

Supplement D. Study 1 Additional Analyses

Exploratory Analyses

Collective Empowerment

Considering the results of the Empowerment measure EFA, we also wanted to assess the model with each subscale separately. Thus, Collective Efficacy and General Empowerment replaced Collective Empowerment in two separate Model 81 analyses.

**Model 81: Collective Efficacy.** Overall, the model does not differ from the Model 81 analysis found in the main manuscript. A positive indirect effect of Condition on Collective Action Intention as sequentially mediated by Collective Validation and General Empowerment (β=0.01, 95% CI (0.00, 0.02) was found, which is consistent with the findings of Model 81 in the main manuscript. Interestingly, a positive indirect effect of Condition on Collective Action Intention mediated by Collective Validation (β=0.04, 95% CI (0.00, 0.09) was also found in this model, which differs from the main analysis.

Table D.1. Correlation matrix for all variables.

|  | Mean (SD) | Condition | Collective Validation | Collective Emotions | Collective  Efficacy |
| --- | --- | --- | --- | --- | --- |
| Collective Validation | 4.3 (.7) | .18** |  |  |  |
| Collective Emotions | 4.4 (.7) | .06 | .01 |  |  |
| Collective Efficacy | 4.23 (1.4) | .10* | .16** | .002 |  |
| Collective Action Intentions | 5.6 (1.2) | -.01 | .11* | .26** | .12** |

* Correlation significant at the 0.05 level

** Correlation significant at the 0.01 level

Table D.2. Direct effects of model 81 with Collective Efficacy.

|  | **Collective Efficacy** | | | | |
| --- | --- | --- | --- | --- | --- |
|  |  | | | **95% CI** | |
|  | ***B*** | ***SE*** | ***p*** | ***LL*** | ***UL*** |
| Condition | 0.20 | 0.12 | .11 | -0.05 | 0.45 |
| Feelings of Collective Validation | **0.31** | **0.1** | **<.01** | **0.12** | **0.50** |
| Collective Anger & Contempt | - | - | - | - | - |
| Collective Efficacy | - | - | - | - | - |
| Constant | 2.60 | 0.42 | <.001 | 1.78 | 3.44 |
|  | *R*^2^ = 0.03  *F*(2,517) = 7.69, *p* < .001 | | | | |

|  | **Collective Action Intent** | | | | |
| --- | --- | --- | --- | --- | --- |
|  |  | | | **95% CI** | |
|  | ***B*** | ***SE*** | ***p*** | ***LL*** | ***UL*** |
| Condition | -0.12 | 0.10 | .23 | -0.32 | 0.08 |
| Feelings of Collective Validation | **0.17** | **0.08** | **.03** | **0.02** | **0.32** |
| Collective Anger & Contempt | **0.44** | **0.07** | **<.001** | **0.30** | **0.57** |
| Collective Efficacy | **0.02** | **0.04** | **<.01** | **0.02** | **0.16** |
| Constant | 2.73 | 0.46 | <.001 | 1.83 | 3.63 |
|  | *R*^2^ = 0.09  *F*(4,515) = 13.21, *p* < .001 | | | | |

.23***

.31**

-.01

.44***

.02**

Condition (Cancel Culture vs. Control)

Feelings of Collective Validation

Collective Action Intention

Collective Efficacy

Collective Anger & Contempt

Figure D.1. Collective Efficacy Model Results.

* p<.05; ** p<.01; *** p<.001

**Model 81: General Empowerment.** Once again, the overall model does not differ from the Model 81 analysis found in the main manuscript. A positive indirect effect of Condition on Collective Action Intention as sequentially mediated by Collective Validation and General Empowerment (β=0.04, 95% CI (0.02, 0.08)) was found, which is consistent with the findings of Model 81 in the main manuscript.

Table D.3. Correlation matrix for all variables.

|  | Mean (SD) | Condition | Collective Validation | Collective Emotions | General Empowerment |
| --- | --- | --- | --- | --- | --- |
| Collective Validation | 4.3 (.7) | .18** |  |  |  |
| Collective Emotions | 4.4 (.7) | .06 | .01 |  |  |
| General Empowerment | 5.2 (1.1) | .03 | .30** | .12** |  |
| Collective Action Intentions | 5.6 (1.2) | -.01 | .11* | .26** | .39** |

* Correlation significant at the 0.05 level

** Correlation significant at the 0.01 level

Table D.4. Direct effects of model 81 with General Empowerment.

|  | **General Empowerment** | | | | |
| --- | --- | --- | --- | --- | --- |
|  |  | | | **95% CI** | |
|  | ***B*** | ***SE*** | ***p*** | ***LL*** | ***UL*** |
| Condition | -.06 | 0.09 | .55 | -0.24 | 0.13 |
| Feelings of Collective Validation | **0.51** | **0.07** | **<.01** | **0.37** | **0.65** |
| Collective Anger & Contempt | - | - | - | - | - |
| General Empowerment | - | - | - | - | - |
| Constant | 3.07 | 0.32 | <.001 | 2.45 | 3.69 |
|  | *R*^2^ = 0.09  *F*(2,517) = 25.86, *p* < .001 | | | | |

|  | **Collective Action Intent** | | | | |
| --- | --- | --- | --- | --- | --- |
|  |  | | | **95% CI** | |
|  | ***B*** | ***SE*** | ***p*** | ***LL*** | ***UL*** |
| Condition | -0.07 | 0.09 | .44 | -0.26 | 0.11 |
| Feelings of Collective Validation | **0.00** | **0.07** | **.97** | **-.14** | **0.11** |
| Collective Anger & Contempt | **0.36** | **0.07** | **<.001** | **0.23** | **0.49** |
| General Empowerment | **0.38** | **0.04** | **<.01** | **0.02** | **0.16** |
| Constant | 2.10 | 0.43 | <.001 | 1.25 | 2.95 |
|  | *R*^2^ = 0.09  *F*(4,515) = 13.21, *p* < .001 | | | | |

.23***

.51**

-.01

.36***

.38**

Condition (Cancel Culture vs. Control)

Feelings of Collective Validation

Collective Action Intention

General Empowerment

Collective Anger & Contempt

Figure D.2. General Empowerment Model Results.

* p<.05; ** p<.01; *** p<.001

Collective Emotions

While Feelings of Collective Validation and Collective Empowerment sequentially mediated the relationship between Condition and Collective Action Intentions, as predicted, Feelings of Collective Validation and Collective Anger and Contempt did not. Collective Anger and Contempt have a positive relationship with Collective Action Intentions, as expected, which is consistent with previous research. However, Feelings of Collective Validation do not have a significant relationship with Collective Anger and Contempt. It is possible, though, that Feelings of Collective Validation elicited by cancel culture impact Collective Anger and Contempt independently.

Therefore, two exploratory analyses using SPSS PROCESS Model 6 assess the potential serial mediation pathways in Figure D.3 below for Collective Anger and Contempt, independently.

Feelings of Collective Validation

Collective Action Intentions

Condition (Control vs. Cancel Culture)

Collective Anger

Feelings of Collective Validation

Collective Action Intentions

Condition (Control vs. Cancel Culture)

Collective Contempt

Figure D.3. Exploratory Anger Model 6 (top) and Contempt Model 6 (bottom).

Results show no direct effect of Condition or Feelings of Collective Validation on Collective Contempt or Anger. The indirect pathway between Condition, Feelings of Collective Validation, Collective Anger, and Collective Action Intentions was not significant, as was the indirect pathway between Condition, Feelings of Collective Validation, Contempt, and Collective Action. There was also no indirect effect of either pathway: a) Condition, Anger, and Collective Action Intentions nor b) Condition, Contempt, and Feelings of Collective Validation. Therefore, Condition and Feelings of Collective Validation seem to have no impact on Collective Anger or Contempt indicating that the emotional pathway to Collective Action Intentions is distinct from the Feelings of Collective Validation and Collective Empowerment pathway to Collective Action Intentions.

Table D.5. Direct effects of the exploratory Collective Anger Model 6.

|  | **Feelings of Collective Validation** | | | | |
| --- | --- | --- | --- | --- | --- |
|  |  | | | **95% CI** | |
|  | ***B*** | ***SE*** | ***p*** | ***LL*** | ***UL*** |
| Condition | 0.23 | 0.06 | <.001 | 0.12 | 0.34 |
| Feelings of Collective Validation | - | - | - | - | - |
| Collective Anger | - | - | - | - | - |
| Constant | 3.92 | 0.09 | <.001 | 3.74 | 4.10 |
|  | *R*^2^ = 0.03  *F*(1,518) = 16.28, p < .001 | | | | |

|  | **Collective Anger** | | | | |
| --- | --- | --- | --- | --- | --- |
|  |  | | | **95% CI** | |
|  | ***B*** | ***SE*** | ***p*** | ***LL*** | ***UL*** |
| Condition | 0.12 | 0.07 | .07 | -0.01 | 0.25 |
| Feelings of Collective Validation | -0.02 | 0.05 | .74 | -0.12 | 0.08 |
| Collective Anger | - | - | - | - | - |
| Constant | 4.31 | 0.22 | <.001 | 3.87 | 4.74 |
|  | *R*^2^ = 0.06  *F*(2,517) = 1.63, *p* = 0.197 | | | | |

|  | **Collective Action Intentions** | | | | |
| --- | --- | --- | --- | --- | --- |
|  |  | | | **95% CI** | |
|  | ***B*** | ***SE*** | ***p*** | ***LL*** | ***UL*** |
| Condition | -0.11 | 0.10 | .28 | -0.31 | 0.09 |
| Feelings of Collective Validation | 0.20 | 0.08 | <.01 | 0.05 | 0.35 |
| Collective Anger | 0.40 | 0.08 | <.001 | 0.26 | 0.53 |
| Constant | 3.14 | 0.45 | <.001 | 2.26 | 4.01 |
|  | *R*^2^ = 0.07  *F*(3,516) = 13.78, *p* < .001 | | | | |

Table D.6. Direct effects of the exploratory Collective Contempt Model 6.

|  | **Feelings of Collective Validation** | | | | |
| --- | --- | --- | --- | --- | --- |
|  |  | | | **95% CI** | |
|  | ***B*** | ***SE*** | ***p*** | ***LL*** | ***UL*** |
| Condition | 0.23 | 0.06 | <.001 | 0.12 | 0.34 |
| Feelings of Collective Validation | - | - | - | - | - |
| Collective Contempt | - | - | - | - | - |
| Constant | 3.92 | 0.09 | <.001 | 3.74 | 4.10 |
|  | *R*^2^ = 0.03  *F*(1,518) = 16.28, p < .001 | | | | |

|  | **Collective Contempt** | | | | |
| --- | --- | --- | --- | --- | --- |
|  |  | | | **95% CI** | |
|  | ***B*** | ***SE*** | ***p*** | ***LL*** | ***UL*** |
| Condition | 0.06 | 0.07 | .36 | -0.07 | 0.19 |
| Feelings of Collective Validation | 0.01 | 0.05 | .88 | -0.09 | 0.11 |
| Collective Contempt | - | - | - | - | - |
| Constant | 4.32 | 0.22 | <.001 | 3.89 | 4.75 |
|  | *R*^2^ = 0.002  *F*(2,517) = 0.48, *p* = 0.62 | | | | |

|  | **Collective Acton Intent** | | | | |
| --- | --- | --- | --- | --- | --- |
|  |  | | | **95% CI** | |
|  | ***B*** | ***SE*** | ***p*** | ***LL*** | ***UL*** |
| Condition | -0.09 | 0.10 | .39 | -0.29 | 0.11 |
| Feelings of Collective Validation | 0.19 | 0.08 | .01 | 0.04 | 0.34 |
| Collective Contempt | 0.41 | 0.07 | <.001 | 0.28 | 0.55 |
| Constant | 3.06 | 0.45 | <.001 | 2.18 | 3.94 |
|  | *R*^2^ = 0.08  *F*(3,516) = 14.56, *p* < .001 | | | | |

Interestingly, the results show a significant positive pathway between Feelings of Collective Validation and Collective Action Intentions in both the Collective Anger and Collective Contempt Model 6 analyses, but not in the predicted Model 81 analysis. This indicates that when Collective Empowerment is excluded, the relationship between Feelings of Collective Validation and Collective Action Intentions appears as a direct effect. It is also possible that these emotions serve as covariates or moderators.

**Models 1 and 4.** To test this, three additional exploratory analyses were conducted: 1) SPSS PROCESS Model 1 with Collective Anger as a moderator, 2) Model 1 with Collective Contempt as a moderator and 3) Model 4 with Collective Anger and Contempt as covariates. See Figure D.4 for details.

Feelings of Collective Validation

Collective Action Intentions

Collective Anger/

Contempt

Collective Anger/

Contempt

Episode of Cancel Culture

Feelings of Collective Validation

Collective Action Intentions

Figure D.4. Exploratory Model 1 and Model 4 for Anger and Contempt.

In Model 1 (top), Collective Anger and Collective Contempt will be tested as separate moderators on the Feelings of Collective Validation and Collective Action Intentions pathway. In Model 4 (bottom), Collective Anger and Collective Contempt will be included as covariates in a mediation analyses.

**Model 1: Collective Anger.** The interaction between Feelings of Collective Validation and Collective Anger was found to be non-significant (β=-0.09, *SE*=0.09, 95% CI (-0.27, 0.10), *p*=.35). The conditional effect of Feelings of Collective Validation on Collective Action Intentions was significant (β=-0.19, *SE*=0.08, 95% CI (-0.05, 0.34), *p*=.01). Therefore, the results do not indicate Collective Anger as a moderator of the relationship between Feelings of Collective Validation and Collective Action Intentions.

**Model 1: Collective Contempt.** The interaction between Feelings of Collective Validation and Collective Contempt was found to be non-significant (β=-0.09, *SE*=0.10, 95% CI (-0.28, 0.10), *p*=.37). The conditional effect of Feelings of Collective Validation on Collective Action Intention was significant (β=-0.19, *SE*=0.08, 95% CI (-0.04, 0.34), *p*=.01). Therefore, the results do not indicate Contempt as a moderator of the relationship between Feelings of Collective Validation and Collective Action Intention.

**Model 4: Anger and Contempt Covariates.** As evident in Tables D.4 and D.5, Collective Anger and Contempt do not meaningfully impact the relationship between Condition, Feelings of Collective Validation, and Collective Action Intent. Therefore, these results imply that Collective Anger and Contempt, may exist as a pathway to Collective Action Intentions separate from Feelings of Collective Validation and may have a unique, unexplored relationship with cancel culture.

Table D.7. Direct effects of exploratory Model 4 with Anger and Contempt covariates.

|  | **Feelings of Collective Validation** | | | | |
| --- | --- | --- | --- | --- | --- |
|  |  | | | **95% CI** | |
|  | ***B*** | ***SE*** | ***p*** | ***LL*** | ***UL*** |
| Condition | **0.24** | **0.06** | **<.001** | **0.12** | **0.35** |
| Feelings of Collective Validation | - | - | - | - | - |
| Anger | -0.07 | 0.08 | .36 | -0.22 | 0.08 |
| Contempt | 0.07 | 0.08 | .38 | -0.08 | 0.22 |
| Constant | 3.92 | 0.10 | <.001 | 3.54 | 4.31 |
|  | *R*^2^ = 0.03  *F*(3,516) = 5.71, p < .001 | | | | |

|  | **Collective Action Intentions** | | | | |
| --- | --- | --- | --- | --- | --- |
|  |  | | | **95% CI** | |
|  | ***B*** | ***SE*** | ***p*** | ***LL*** | ***UL*** |
| Condition | -0.10 | 0.10 | .33 | -0.30 | 0.10 |
| Feelings of Collective Validation | 0.20 | 0.08 | .01 | 0.04 | 0.35 |
| Anger | 0.17 | 0.13 | .19 | -0.09 | 0.43 |
| Contempt | **0.26** | **0.13** | **.05** | **0.00** | **0.52** |
| Constant | 2.97 | 0.45 | <.001 | 2.08 | 3.86 |
|  | *R*^2^ = 0.08  *F*(4,515) = 11.36, p < .001 | | | | |

Table D.8. Direct effects of exploratory Model 4 without Anger and Contempt covariates.

|  | **Feelings of Collective Validation** | | | | |
| --- | --- | --- | --- | --- | --- |
|  |  | | | **95% CI** | |
|  | ***B*** | ***SE*** | ***p*** | ***LL*** | ***UL*** |
| Condition | 0.23 | 0.06 | <.001 | 0.12 | 0.34 |
| Feelings of Collective Validation | - | - | - | - | - |
| Constant | 3.92 | 0.09 | <.001 | 3.74 | 4.10 |
|  | *R*^2^ = 0.03  *F*(1,518) = 16.28, p < .001 | | | | |

|  | **Collective Action Intentions** | | | | |
| --- | --- | --- | --- | --- | --- |
|  |  | | | **95% CI** | |
|  | ***B*** | ***SE*** | ***p*** | ***LL*** | ***UL*** |
| Condition | -0.06 | 0.10 | .55 | -0.27 | 0.14 |
| Feelings of Collective Validation | **0.20** | **0.08** | **.01** | **0.04** | **0.35** |
| Constant | 3.92 | 0.35 | <.001 | 4.15 | 5.53 |
|  | *R*^2^ = 0.01  *F*(2,517) = 3.08, p = 0.04 | | | | |

Supplement E. Study 2 Materials

Manipulation

Cancel Culture Condition

**The scenario in this article is true and occurred in the Metro Vancouver (Canada) area. While you read this article, imagine that this scenario occurred in your neighbourhood.**

Store Owner Facing Backlash for Anti-Asian Racism/Xenophobia

James Fitz, owner of a small Vancouver-based pet supply business, and one of his employees, are being criticized by the community for unleashing a racist and xenophobic rant directed at a customer.

The customer told reporters that the incident began when he asked the employee about a dog food return. “That’s when it turned ugly. He looked at me intensely – I think he was trying to see my eyes because I had a mask and a hat on.”

Then the employee said, “You mean you actually have dogs as pets?”

At first, the customer was confused, “But then he and the owner laughed and said, ‘You know, this is all because of you people eating animals that you shouldn’t be eating.’ That’s when I understood what was happening – that he was talking about me being East Asian, and talking about Covid.” Then he explained how the employee and owner continued to yell at him – racial slurs included – about Covid and how it is Asian people’s fault for “unleashing this on everybody”.

Another customer, who filmed the incident, said “The man was trying to leave the store and the owner told him, ‘you should stay in your own country and not bring your diseases with you.’ Then he spat at him.”

The video and a description of the incident was shared online. Within three days, the post had 2200 likes and has been shared over 300 times by the Vancouver community and beyond. Twitter and Instagram users from the Metro Vancouver community have responded with calls for boycott of the pet supply store, legal repercussions, and #FuckFitzsPetSupply. Posters reading “racist” and “don’t shop here” have been posted outside the store in warning to customers. The community has also identified the employee as a student from a nearby university and have called on the university to take action.

Other members of the community are also condemning Fitz and the employee’s behaviour. “This is deplorable, especially during a time when we should be working together” says the owner of a nearby clothing store, “East-Asian people are right to expect better from their neighbours. I’m pleased to report that fewer shoppers are going to the store.”

Other community members agree. Messages from various community groups online state, “They hide behind their smiles and niceties, but when a disaster happens, we see their racism. It has always been there.” This is referring to the long history of anti-Asian racism in Canada from the building of the transnational railroad to the more recent “foreign buyers tax”. Yet, it is only the recent 717% increase in anti-Asian hate crimes since the start of the Covid-19 pandemic that has caused Vancouver to be labelled the ‘anti-Asian hate crime capital of North America’. “It’s time for racists in this community to face the consequences of their actions. If this bigot loses his business because of this, so be it” writes another group online.

It seems these aren’t the only community members to think so. A petition has been created calling for the police to investigate the store owner and employee and charge them with a hate crime, while the university community has also circulated a petition and planned a campus rally calling for the immediate expulsion of the employee from his program. While police have yet to comment on their plans for investigation, the pet supply store will temporarily close next week, and both Fitz and the employee have disabled their social media.

Control Condition

**The scenario in this article is true and occurred in the Metro Vancouver (Canada) area. While you read this article, imagine that this scenario occurred in your neighbourhood.**

Anti-Asian Racism/Xenophobia on the Rise Following Covid-19

James Fitz, owner of a small Vancouver-based pet supply business, and one of his employees, unleashed a racist and xenophobic rant directed at a customer.

The customer told reporters that the incident began when he asked the employee about a dog food return. “That’s when it turned ugly. He looked at me intensely – I think he was trying to see my eyes because I had a mask and a hat on.”

Then the employee said, “You mean you actually have dogs as pets?”

At first, the customer was confused, “But then he and the owner laughed and said, ‘You know, this is all because of you people eating animals that you shouldn’t be eating.’ That’s when I understood what was happening – that he was talking about me being East Asian, and talking about Covid.” Then he explained how the employee and owner continued to yell at him – racial slurs included – about Covid and how it is Asian people’s fault for “unleashing this on everybody”.

Another customer, who filmed the incident, said “The man was trying to leave the store and the owner told him, ‘you should stay in your own country and not bring your diseases with you.’ Then he spat at him.”

The video and a description of the incident was shared online. Police have yet to comment on their plans for investigation, even after releasing a recent report showing that a 717% increase in anti-Asian hate crimes since the start of the Covid-19 pandemic has caused Vancouver to be labelled the ‘anti-Asian hate crime capital of North America’.
