# Supplementary material for: Cancel culture can be collectively validating for groups experiencing harm
Source: Front Psychol. 2023 Jul 20;14:1181872. doi: 10.3389/fpsyg.2023.1181872 (PMC10399695; doi:10.3389/fpsyg.2023.1181872)
Supplement: Supplementary file 2 [file Data_Sheet_2.docx]

**APPENDIX A: Collective Validation Measures**

**Study 1**

*After reading this article and imagining that these events happened within the SFU community*, please indicate the extent to which you agree or disagree with each of the following statements.

1. I feel that the university community ignores women. (R)
2. I feel seen by the university community.
3. I feel that women are being sufficiently heard by the university community.
4. I feel silenced by the university community.
5. (R)
6. The experiences of harm faced by women are recognized by the university community.
7. The experiences of harm faced by women are affirmed in the university community.
8. I feel that the experiences of women are not acknowledged by the university community. (R)
9. The university community understands that misogyny is harmful to women.
10. I feel that my emotional response to sexism and misogyny is shared by others in the university community.
11. I would feel that my perception of experiences with sexism are exaggerated. (R)
12. The university community response makes me feel justified in demanding reparations for harm done to women.
13. I feel unsupported by the university community. (R)
14. I feel uncomfortable demanding justice. (R)
15. I feel supported by the university community in demanding reparations.
16. I feel that the outcome of the scenario was just.
17. Women are right to be angry at misogynistic men.
18. It is right to feel contempt for misogynistic men.
19. My contempt for the men in this scenario is not justified. (R)
20. Misogynistic men deserve to be expelled from the university community.
21. My emotional response to sexism and misogyny is justified.
22. The experiences of women are validated by the university community.
23. As a woman, I feel validated by the university community.
24. I feel that my emotions about the situation were invalid. (R)
25. My emotional responses to sexism and misogyny are valid.

**Study 2**

**Still imagining that the scenario occurred in your community, please complete the following questions.**

***After reading this article and imagining that these events happened within your community***, please indicate the extent to which you agree or disagree with each of the following statements.

1. I feel that this community ignores East Asian people.
2. If I were in this scenario, I would feel seen by the community.
3. I feel that East Asian people are being sufficiently heard by this community.
4. If I were in this scenario, I would feel silenced by the community.
5. The experiences of harm faced by East Asian people are recognized by the community.
6. The experiences of harm faced by East Asian people are affirmed in the community.
7. I feel that the experiences of East Asian people are not acknowledged by the community.
8. This community understands that racism is harmful to East Asian people.
9. I feel that my emotional response to racism and xenophobia is shared by others in the community.
10. If I were in this scenario, I would feel that my perception of experiences with racism are exaggerated.
11. The community response makes me feel justified in demanding reparations for harm done to East Asian people.
12. If I were in this scenario, I would feel unsupported by the community.
13. If I were in this scenario, I would feel uncomfortable demanding justice.
14. If I were in this scenario, I would feel supported by the community in demanding reparations.
15. I feel that the outcome of the scenario was just.
16. East Asian people are right in their anger toward racist people.
17. It is right to feel contempt for racist people.
18. My contempt for the racist people in this scenario is not justified.
19. Racist people deserve to be expelled from the community.
20. My emotional response to racism and xenophobia is justified.
21. The experiences of East Asian people are validated by the community.
22. As an East Asian person, I feel validated by the community.
23. If I were in this scenario, I would feel that my emotions about the situation were invalid.
24. My emotional response to racism and xenophobia is valid.
